# 不同组织亚型不同基因型同时性多原发肺癌（附1例病例报道）

**DOI:** 10.3779/j.issn.1009-3419.2017.12.11

**Published:** 2017-12-20

**Authors:** 欣 李, 智荣 章, 毅立 傅, 劲柏 苗, 滨 胡

**Affiliations:** 100020 北京，首都医科大学附属北京朝阳医院胸外科 Department of Thoracic Surgery, Beijing Chaoyang Hospital, Capital Medical University, Beijing 100020, China

**Keywords:** 多原发癌, 病理亚型, EGFR, 肺肿瘤, Multiple primary cancer, Pathological subtype, EGFR, Lung neoplasms

## Abstract

随着低剂量螺旋计算机断层扫描（computed tomography, CT）广泛应用于早期肺癌筛查，诊断出的双肺同时多发性病变的病例数逐年增多，且大多数病例最后证实为同时性多原发肺癌（synchronous multiple primary lung cancer, SMPLC）。既往研究结果显示SMPLC的发病率约为0.2%-8%（穿刺研究结果为3.5%-14%）。目前对于MPLC的诊断，大多依据Martini-Melamed的诊断标准：SMPLC：①肺癌部位各异，彼此孤立；②组织学类型不同；③组织学类型相同时，位于不同肺段、肺叶、不同侧肺，由不同的原位癌起源，肺癌共同的淋巴引流部位无癌肿，确立诊断时无肺外转移。异时性多原发肺癌（metachronous multiple primary lung cancer, MMPLC）：①组织学类型不同；②组织学类型相同时，无瘤间期至少2年，或均由不同的原位癌起源，或第二原发癌位于不同肺叶或不同侧肺时，肺癌共同的淋巴引流部位无癌肿，确立诊断时无肺外转移。而各个肿瘤具有独特的病理形态特征为诊断MPLC的要点。此后很多学者在这条标准的基础上进行了不断的修订与丰富，包括国际肺癌研究协会（International Association for the Study of Lung Cancer, IASLC）新版肺腺癌分类及表皮生长因子受体（epidermal growth factor receptor, EGFR）、*K*-*ras*基因突变等，这些内容的增加使多原发癌灶与转移灶的鉴别诊断标准更加合理准确，也同时说明对于MPLC的各个病灶分别进行基因检测具有重要的鉴别和治疗意义。现将我院胸外科术后病理证实为不同组织亚型、不同基因型的同时性四原发肺癌患者诊治情况报道如下。

## Abstract (en)

In recent years, based on low-dose computed tomography (CT) scan developed for physical examination, the number of synchronous multiple primary lung cancer (SMPLC) has gradually increased. The research showed the morbidity of SMPLC up to 0.2%-8%. The current diagnostic criteria of SMPLC is Martini-Melamed criteria. SMPLC: (1) Tumours should be located distantly and separately; (2) Histological types: Different histology; If same histology, they should be located at different segment, lobe or lung and originated from carcinoma insitu. No presence of carcinoma at shared lymphatic drainage. No extrapulmonary metastases at the diagnosis. MMPLC: (1) Different histology; (2) Disease free duration more than 2 years. Originated from carcinoma *in situ* location of second cancer at different lobe or lung. No presence of carcinoma at shared lymphatic drainage. No extrapulmonary metastases at the diagnosis. In 2013 International association for lung cancer research (IASLC) amended and supplemented the Martini-Melamed criteria, by multidisciplinary classification of lung adenocarcinoma, epidermal growth factor receptor (*EGFR*), K-ras added for differential diagnosis. It also suggest that the gene mutation detection for each lesion of SMPLC is especially significant for therapeutic strategy. We herein report the case of a 60-year-old woman diagnosed with SMPLC of four adenocarcinoma and *EGFR*-mutated lesions, who received lung resection for each lesions.

## 1 病例资料

患者女性，60岁，因"咳嗽、发热2月"入住当地医院，行胸片检查发现右肺结节影。未予治疗。于2016年10月8日收入我科。既往高血压病5年、肾功能不全病史4年。无吸烟史。查体：浅表淋巴结未触及肿大，双肺叩诊清音，双肺听诊呼吸音清，未闻及干湿啰音，心脏、腹部、四肢查体未见异常。

肿瘤标志物：细胞角蛋白19片段（cytokeratin-19-fragment, CYFRA21-1）2.13 ng/mL（正常范围：0-2.08 ng/mL），胃泌素释放肽前体（pro-gastrin releasing peptide, ProGRP）52.45 pg/mL（正常范围：0-50.00 pg/mL）。肺功能：一秒用力呼气容积（forced expiratory volume in one second, FEV_1_）2.92 L，FEV_1_% 77.2%，一氧化碳弥散量（diffusion capacity of carbon monoxide, DLCOc SB）7.62 mmol/min/kPa，DLCOc SB% 106.3%；胸部增强计算机断层扫描（computed tomography, CT）：右肺中叶外侧段可见类圆形结节影，大小约2.0 cm×1.8 cm，边缘可见分叶及毛刺；右肺上叶后段可见磨玻璃结节影、亚实性结节影；右肺下叶前基底段可见片状磨玻璃样影；纵隔淋巴结未见肿大。全身骨扫描、腹部超声及脑核磁未见明显异常。

考虑到病变均位于右侧，于2016年10月10日全麻下行胸腔镜（video-assisted thoracoscopic surgery, VATS）右肺中叶切除+右肺上叶后段+右肺下叶楔形切除术+纵隔淋巴结清扫术，手术顺利。术后患者恢复佳顺利出院，后未行辅助化疗并返回当地，术后6个月复诊胸部CT未见肿瘤复发。术后病理及基因检测结果列于图 1。

**1:**
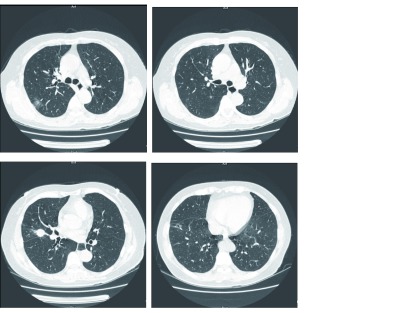
病灶CT影像、病理亚型及基因突变。A：右上肺病灶：*EGFR* 19Del mutation，Acinar（50%），Lepidic（45%），Papillary（5%）；B：右肺上叶病灶；*EGFR* 21-L858R mutation，Acinar（60%），Lepidic（40%）；C：右肺中叶病灶；*EGFR* 19Del mutation，Papillary（55%），Acinar（40%），Lepidic（5%）；D：右肺下叶病灶。*EGFR* 19Del mutation，Papillary（30%），Acinar（20%），Lepidic（50%）。 The CT image, pathologic subtype and mutation of differrent lesions. A: Lesion No.1 in right upper lobe: *EGFR* 19Del mutation, Acinar (50%), Lepidic (45%), Papillary (5%); B: Lesion No.2 in right upper lobe: *EGFR* 21-L858R mutation, Acinar (60%), Lepidic (40%); C: Lesion in right middle lobe: *EGFR* 19Del mutation, Papillary (55%), Acinar (40%), Lepidic (5%); D: Lesion in right lower lobe: *EGFR* 19Del mutation, Papillary (30%), Acinar (20%), Lepidic (50%). CT: computed tomography; EGFR: epidermal growth factor receptor.

## 2 讨论

随着肺癌早期筛查观念的广泛普及与肿瘤诊疗技术的进步，尤其是高分辨率CT的广泛应用，临床诊出的多原发肺癌（multiple primary lung cancer, MPLC）病例数不断增加。MPLC是指在指在同一个体上，发现并被病理证实的多起源的原发肺癌，相同肺叶或不同肺叶多个独立而相互之间无从属关联关系的癌灶。

### 2.1 同时性多原发肺癌（synchronous MPLC, SMPLC）的临床诊断

目前临床上术前对SMPLC的诊断及鉴别主要仍依赖于胸部CT，SMPLC的各癌灶大多具有原发性肺癌的典型CT表现，如边缘毛刺、胸膜牵拉、分叶、血管集束征、密度混杂（如磨玻璃样或实性）、增强窗可见强化等等。另外，初诊时往往定性存在一定困难，CT的随访观察也是鉴别诊断的重要手段，未治的原发肺癌（主病变处理后）常常发展缓慢，而转移灶患者进展迅速，患者的一般情况较差。而肺单发或多发转移瘤多为圆形和椭圆形，边缘一般较光整，很少具有毛刺和胸膜牵拉征象。本病例中此患者的术前CT提示右肺中叶实性结节、右肺上叶后段磨玻璃结节影及亚实性结节边缘带毛刺、右肺下叶前基底段片状磨玻璃样影伴胸膜牵拉，具有原发肺癌的典型影像学表现，故考虑患者存在同时性多原发肺癌可能。临床上SMPLC与肺内多发转移瘤的治疗方法和预后存在着巨大差异，因此术前对SMPLC的鉴别非常重要。对于检查所发现的双肺多发病变，应该仔细评估诊断并慎重制订诊疗方案。避免判定为晚期转移病变而致使部分患者错失手术机会。

对SMPLC的确诊主要依赖于术后的组织病理学、分子生物学和基因检测。1975年Martini和Melamed总结50例多原发癌患者的临床资料，首次提出MPLC的诊断标准^[1, 2]^。此后研究者均在此基础上进行了不断改进。2012年国际肺癌研究协会（International Association for the Study of Lung Cancer, IASLC）已推荐肺腺癌新分类方法可以作为鉴别MPLC的一个因素来考量^[3, 4]^。2013年，美国胸科医师协会（American College of Chest Physicians, ACCP）对多原发癌这一问题进行了更新，改良了Martini标准：（1）①组织学类型相同、原发于不同肺叶；②无N2、N3淋巴结转移；③无全身性转移灶。（2）①组织学类型不同；②基因分子生物学特征不同；③起源不同的原位癌灶。（3）①组织学类型相同；②异时发病，两次发病间隔＞4年；③无全身性转移灶^[5]^。在此报告病例中，右肺中四个癌灶均出现不同的原位癌灶，呈不同的腺癌组织亚型生长；同时*EGFR*基因出现不同的外显子体细胞突变与文献第二种MPLC类型提出的鉴别依据一致。

### 2.2 SMPLC的手术治疗

目前手术治疗SMPLC的手术指征与手术方式的选择依然是困扰胸外科医师的难题。对于多原发肺癌，术前应根据患者一般状况和心肺功能做严格术前检查，影像学检查排除纵膈淋巴结及远处转移的患者应及时行手术治疗，选择合理的术式。（1）同叶双原发或多原发：同期手术多采用肺叶切除；（2）不同肺叶单发病灶：①位于同侧不同肺叶单发病灶：若患者肺功能允可，可采取同期手术，一般较大病灶所在的肺叶行肺叶切除术，小病灶采取亚肺叶切除；若两病灶较小，可采用不同亚肺叶切除，已有研究数据显示对于GGO病灶，这一方法与肺叶切除效果等同。②位于双侧不同肺叶单发病灶：原则上分期手术，2次手术间隔4周-6周，遵循的原则是：先切除中央型肺癌或进展较快的病灶，后切除周围型肺癌；先切除病灶较大者，后切除病灶较小者；即先切除主要影响预后和分期较晚的病变。并在术中获取淋巴结样本以决策术后是否进行辅助化疗。但无论如何，始终应该在安全和根治的两大原则基础上，遵循"两个最大限度"的手术准则：即最大限度切除肿瘤和最大限度保留正常肺组织。（3）双肺多发病灶：内科治疗为主，先剖胸探查获取病理及基因检测结果以选取最佳的治疗措施，如*EGFR*、*ALK*等基因突变阳性，可以选择靶向治疗或化疗。虽然研究表明外科切除后生存明显延长，但并不意味着外科是唯一手段，也有文献^[6]^报道立体定向放疗（stereotactic body radiation therapy, SBRT）也可以作为治疗MPLC的重要治疗手段，尤其适用于一些高龄或心肺功能不能耐受手术的患者。因此，是否选择外科治疗需依据临床实际情况决定。本病例中患者病灶均位于右侧，同时病变均分期较早，均达到根治性切除。

### 2.3 SMPLC的分子生物学检测

需要注意的是，不仅对多原发肺癌的外科处理上按照不同的单原发癌灶的手术原则来切除，术后的基因检测及分子生物学检测等也需要对多病灶逐一检测。2016年的一项研究为129例患者进行了*EGFR*、*BRAF*、*ROS*-*1*、*KRAS*和*ALK*重排基因突变检测，其中87.6%的患者存在各种基因突变，女性SMPLC患者中EGFR突变比例高达72.73%^[7]^，另一项研究^[8]^对相同病理亚型的6例SMPLC患者进行基因检测后确诊了其中5例患者为不同基因型病灶，这对临床多原发肺癌的鉴别和治疗有重要意义。治疗某些肺功能较差而病灶位于不用肺叶的SMPLC患者时，也可以在术前进行穿刺活检并进行基因检测，如果其中一个病灶存在突变，可以将没有基因突变的病灶手术切除，存在突变的病灶进行基因靶向治疗。

### 2.4 SMPLC的预后因素

早期发现诊断和手术切除肿瘤是提高SMPLC疗效的关键。既往文献^[9-11]^报道同时性多原发肺癌术后5年总体生存率为约为34%-60.9%。但由于不同研究存在入组样本量差异较大、诊断标准不一等情况，不同研究结果^[1, 12]^报道的SMPLC术后5年总体生存率相差较大。SMPLC患者术后疗效的相关预后因素也因不同的研究而存在差别。性别、年龄、全肺切除、FEV_1_%、手术方式、辅助化疗、淋巴结状态、是否位于同肺叶等等均是SMPLC术后的独立预后影响因素^[5, 4, 13, 14]^。Tanvetyanon的研究^[15]^表明最大肿瘤直径以及所有肿瘤直径总和是影响患者预后生存的重要因素。本病例术后的远期疗效需要进一步随访明确。

### 2.5 总结

国内目前尚没有多原发肺癌的诊治指南，关于多原发肺癌的研究也较少，仅少数大规模中心有一定诊治经验。首先要提高对多原发肺癌的认识。对于检查所发现的肺部多发病变，应该仔细评估诊断并慎重作出诊疗方案。切不可武断地判定为晚期转移病变而致使部分患者错失手术机会。外科手术治疗能够为SMPLC患者带来很好的生存获益，但是否均应手术治疗以及采取何种的手术方式需依据临床实际情况决定。术后准确鉴别MPLC和转移癌十分重要，因为它影响着疾病分期、治疗决策以及患者预后。尽管目前临床上对多病灶分别进行基因检测价格昂贵，难以广泛应用，但随着分子诊断技术的发展，吸纳更多的基因分子特征，将有助于提供更加准确的鉴别MPLC和转移癌的依据，也必将为MPLC患者提供科学合理的规范化诊治。
